# Investigating the Effect of Encapsulation Processing Parameters on the Viability of Therapeutic Viruses in Electrospraying

**DOI:** 10.3390/pharmaceutics12040388

**Published:** 2020-04-24

**Authors:** Tayo Sanders, Anita Milicic, Eleanor Stride

**Affiliations:** 1 Department of Engineering Science, Institute of Biomedical Engineering, University of Oxford, Old Road Campus Research Building, Headington OX3 7DQ, UK; tayo.sandersii@eng.ox.ac.uk; 2 Nuffield Department of Medicine, The Jenner Institute, University of Oxford, Old Road Campus Research Building, Headington OX3 7DQ, UK; anita.milicic@ndm.ox.ac.uk

**Keywords:** modified vaccinia Ankara, Adenovirus, vaccine delivery, encapsulation, electroporation

## Abstract

The ability of viruses to introduce genetic material into cells can be usefully exploited in a variety of therapies and also vaccination. Encapsulating viruses to limit inactivation by the immune system before reaching the desired target and allowing for controlled release is a promising strategy of delivery. Conventional encapsulation methods, however, can significantly reduce infectivity. The aim of this study was to investigate electrospraying as an alternative encapsulation technique. Two commonly used therapeutic viruses, adenovirus (Ad) and modified vaccinia Ankara (MVA), were selected. First, solutions containing the viruses were electrosprayed in a single needle configuration at increasing voltages to examine the impact of the electric field. Second, the effect of exposing the viruses to pure organic solvents was investigated and compared to that occurring during coaxial electrospraying. Infectivity was determined by measuring the luminescence produced from lysed A549 cells after incubation with treated virus. Neither Ad nor MVA exhibited any significant loss in infectivity when electrosprayed within the range of electrospraying parameters relevant for encapsulation. A significant decrease in infectivity was only observed when MVA was electrosprayed at the highest voltage, 24 kV, and when MVA and Ad were exposed to selected pure organic solvents. Thus, it was concluded that electrospraying would be a viable method for virus encapsulation.

## 1. Introduction

Evolution has honed the ability of viruses to deliver a payload of genetic material to a target cell. Consequently, viral therapies have attracted a great deal of interest for the development of cancer treatments, vaccines, and various gene therapies. In the context of vaccination, viral vectors generate a robust humoral and cell-mediated immune response to the encoded antigen. They also offer a versatile platform that could enable the development of vaccines for infectious diseases, such as malaria, that have seen little success with traditional vaccine development strategies [1]. Moreover, viral vectored vaccines utilising non-replicating adenovirus (Ad) and modified vaccinia Ankara (MVA) have demonstrated excellent safety and immunogenicity, as well as good efficacy against flu, malaria, respiratory syncitial virus (RSV) and Ebola infection. [2,3,4]. In a heterologous prime-boost approach, the Ad vector elicits potent antibody responses against the encoded antigen and MVA contributes the important T cell component in both elderly adults and infants [4]. 

One of the primary challenges in fully realizing the benefits of viral therapies is ensuring that the delivered virus prevails over pre-existing immunity. While viral vectors can be delivered unmodified, some vectors, such as adenovirus, can be rendered largely ineffective because of the prevalence of neutralizing antibodies for several Ad strains in hosts [5]. Alternative strains, such as those derived from simians, with sparse pre-existing immunity in the target population, outperform other strains, but only when pre-existing immunity is a factor [6,7]. To enhance the longevity of more efficacious therapeutic virus strains in the body, a number of studies have explored ‘stealthing’ viruses by attaching various polymers such as polyethylene glycol (PEG) to the viral capsid or envelope [8,9]. This technique has demonstrated success in reducing viral inactivation by the innate immune response and allows for retargeting. The covalent bonding process, however, is slow and can result in decreased infectivity of the virus [10]. If retargeting is not desired and longer in vivo survival is required, the virus can be encapsulated using lipids or biodegradable polymers, such as alginate or poly(lactic-co-glycolic acid) (PLGA). These protect the virus while preserving its natural tropism and infectivity until it is released [11,12,13]. With this strategy, notable applications such as viral vector-based vaccines that need only be administered once could be realized, as the booster doses can be delivered in the biodegradable vehicle alongside the prime dose for delayed release. In addition to facilitating global vaccination coverage and compliance, single-dose vaccines would have a particularly important role in stemming epidemics of outbreak pathogens. 

There are multiple techniques available for therapeutic encapsulation. However, although many of these techniques are sufficient for producing drug loaded microparticles, the sensitive nature of viral vectors necessitates careful consideration and control of processing conditions [14,15]. Conventional encapsulation methods, e.g., emulsion solvent evaporation/extraction, coacervation, membrane emulsification, and spray drying, have repeatedly been shown to significantly degrade complex molecules such as proteins through exposure to harsh organic solvents and high mechanical stresses [15]. Hence it is necessary to employ a microencapsulation method that preserves virus integrity and affords greater control over the antigen release profile. Microfluidic methods avoid the need for high temperatures and pressures and offer exceptional control over particle characteristics [16]. Novel microfabrication techniques have also been developed specifically for vaccine encapsulation [17]. Generating particles at a sufficient scale, however, requires parallel operation of a very large number of devices and this has hindered widespread industrial translation. A further technique that also offers both mild processing conditions and good control over particle characteristics but may be more readily scalable [18] is electrohydrodynamic processing or electrospraying. In this technique, a strong electric field is used to deform a fluid meniscus into a cone-jet to produce small, uniform droplets from an electrified liquid in a capillary [19]. 

Electrospraying has been studied extensively in biomedical research for its ability to form droplets containing sensitive biological material such as cells and to produce drug-loaded particles with a narrow size distribution [20,21]. In 2003, core-shell structures were obtained by coaxially aligning two capillaries in an electrospraying system [22]. Since then, coaxial electrospraying has demonstrated successful encapsulation and controlled release of proteins that retain high levels of bioactivity after processing [23]. However, whilst it is known that electrospraying can be used with proteins and cells with little or no effect on bioactivity, there has been very limited investigation of its use with viruses [23,24]. Electric fields of the magnitude required for electrospraying (typically kV) could potentially affect viral viability through reactive oxygen species (ROS) generation and/or irreversible electroporation. In a standard electrospraying setup in which corona discharge and dielectric breakdowns are avoided, ROS, specifically ozone and hydrogen peroxide, are generated in vanishingly small quantities [25]. Even so, biomolecules that are especially susceptible to oxidation and are electrosprayed in solution at sub mg/mL concentrations can undergo substantial denaturation via heavy metal-catalysed oxidation [25]. Similarly, the electric field may itself damage the virus. To the best of the authors’ knowledge, the first report of inactivating microorganisms, namely bacteria, with strong electric fields dates back to 1967 [26]. The mechanism of inactivation was more fully understood later as irreversible electroporation, wherein a membrane acting like a capacitor becomes charged and induces a permeabilizing transmembrane potential that either increases osmotic potential to the point of lysis or ruptures the membrane outright [27]. The membrane radius is an important factor, as the magnitude of the transmembrane potential increases with the radius at a constant field strength [28]. Moreover, it is important to consider the ability of the material being encapsulated to respond to changes in the microenvironment. For instance, cells can have complex membrane machinery in the form of protein ion pumps that allows for the active regulation of ion balance and eukaryotic cells employ rGSH to alleviate oxidative stress [29]. Viruses generally lack the capacity to respond to osmotic and oxidative stress with the same sophistication. 

A further concern is that using electrospraying to encapsulate material in a biodegradable polymer requires the use of an organic solvent [30]. The risk of toxicity from the final product can be mitigated by suitable solvent extraction methods, and in the case of electrospraying this is simply through evaporation from the jet during particle formation. The direct effect of the solvent on the integrity of the material to be encapsulated must also be considered, however. In particular, organic solvents are widely used in the destruction of viruses [31].

The aim of this study was to determine whether electrospraying could provide a viable method for virus encapsulation. In particular, the effect of the very high electric fields and/or the solvents typically upon viral infectivity were examined. To this end, a coaxial electrospraying system was designed and configured, and the effect of different processing parameters, particularly electric field strength and solvent exposure, on the viability of two widely used therapeutic viruses, Ad and MVA, was tested. 

## 2. Materials and Methods 

### 2.1. Materials

Human adenovirus type 5 with E1/E3 deletions and a CMV-driven luciferase gene inserted into the E1 region was purchased from The Native Antigen Company (AD001, Oxford, UK). Firefly luciferase recombinant MVA was provided by the Viral Vector Core Facility at the Jenner Institute, University of Oxford, Oxford, UK. Reduced glutathione (rGSH), dichloromethane (DCM), propylene glycol phenyl ether (PPh GE), propylene carbonate (PC), benzyl acetate, anisole, triacetin, and toluene were purchased from Sigma-Aldrich, UK. Cell culture, luciferase, bicinchoninic acid (BCA), and 3-(4,5-dimethylthiazol-2-yl)-5(3-carboxymethony-phenol)-2-(4-sulfophenyl)-2H-tetrazolium (MTS) assays were completed with the following reagents: human adenocarcinoma alveolar basal epithelial A-549 cells (European Collection of Cell Cultures), Dulbecco’s modified Eagle medium (DMEM) (D5546, Sigma-Aldrich, Gillingham, UK), fetal bovine serum (FBS; Life Technologies, Paisley, UK), phosphate-buffered saline (PBS; P5493-1L, Sigma-Aldrich, Gillingham, UK), trypsin-ethylenediaminetetraacetic acid (EDTA) (ThermoFisher, Loughborough, UK), trypan blue (ThermoFisher, Loughborough, UK), luciferase assay system (E1501, Promega, Southampton, UK), reporter lysis buffer 5x (E3971, Promega, Southampton, UK) consisting of 125 mM tris(hydroxymethyl)aminomethane (pH 7.8), 10 MM EDTA, 10 MM Dithiothreitol, 50% glycerol and 5% Triton® X-100, BCA) protein assay kit (Sigma-Aldrich, Gillingham, UK), and CellTiter 96® aqueous one solution cell proliferation assay (G3582, Promega, Southampton, UK).

### 2.2. Electrospraying Rig and Operation

The electrospraying system was designed to accommodate electrospraying with a single or multi-needle assembly in an enclosure with a controlled atmosphere. Wires connecting the voltage source to the needle and ground electrode were routed through holes with an adjustable diameter on the side of the enclosure. Fluid tubing was routed in a similar fashion. A high-speed camera was included to enable real time monitoring of the liquid jet and to confirm its stability during processing. The camera was affixed to a solid steel stand with an adjustable mount and located directly outside the front of the enclosure to allow for visualization while protecting the camera and lens.

For these experiments, a coaxial two-needle stainless steel assembly was used. The outermost needle had an internal diameter of 1.07 mm. The central needle had an internal diameter of 0.34 mm. The outer needle carried the appropriate solvent solution, and the inner needle contained the viral solution. The needles were not electrically insulated from each other, and thus held at the same potential during coaxial electrospraying. When a single jet was desired, the inner needle was blocked, and flow was restricted to the outer needle.

Liquid was supplied to the needle assembly via polytetrafluoroethylene (PTFE) tubing to limit solvent-induced swelling and degradation as much as possible. Syringes containing the aqueous solution of interest were mounted on a Harvard syringe pump (Infuse/Withdraw PHD 2000 programmable dual syringe pump, Harvard Apparatus Ltd, Edenbridge, UK) to control the flow rate of solution to the needle assembly. The capacity of the syringes used for the solutions was 5 mL unless otherwise stated. When coaxial electrospraying was performed, a SGE Gas Tight Luer Lock Syringe (Sigma-Aldrich, Gillingham, UK) mounted on a second syringe pump (Cole-Parmer Syringe Pump, Infuse/Withdraw Programmable, Touchscreen Control WZ-74905-04, Cole-Palmer Instrument Company, Eaton Socon, UK) was used so that both inner and outer flow could be controlled separately.

**Figure 1:**
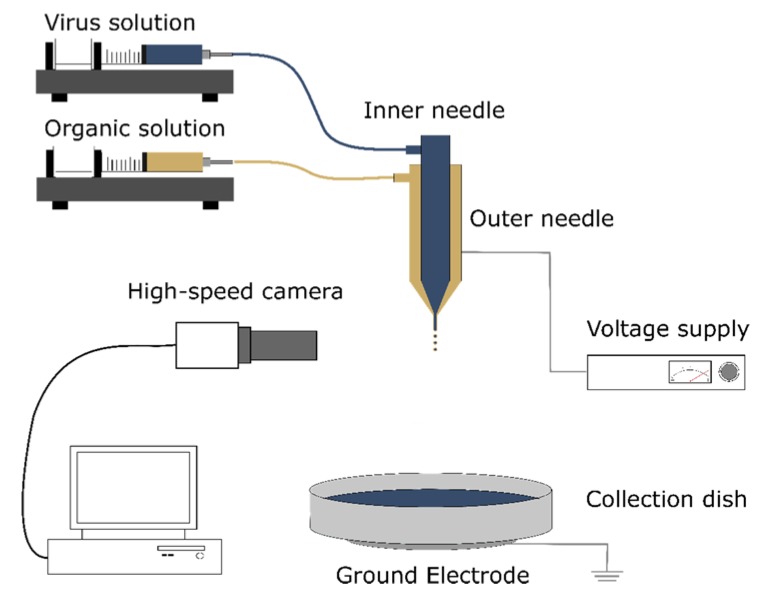
Schematic of the electrospraying rig used for both single and co-axial needle processing.

A sterile aluminium dish was placed on top of the ground electrode 7.25 cm away and was used to collect electrosprayed solutions. PBS (pH 7.4) was used as the base for all electrosprayed solutions unless otherwise stated. The enclosure was sealed, and nitrogen (N_2_) flow was established to eliminate oxygen and thereby avoid the risk of solvent ignition. One mL of the appropriate virus solution was taken up in a sterile syringe. A high voltage DC power supply (PS/FC03P04 12w, Glassman Europe Limited, Bramley, UK) with an output voltage range of 0–30 kV and a current range of 0–4 mA was connected to the needle and ground electrode using alligator clips to create an electric field suitable for electrospraying. After turning on the power supply and setting it to the appropriate voltage, 5 mL of the virus solution was pumped through the needle at a constant rate of 10 µL/min. After the solution had been collected, the tubing and needle assembly were rinsed by flushing through 10 mL of ethanol, followed by 10 mL of PBS. The lowest applied voltage used, 8 kV, marked the average onset of a cone-jet for a variety of solvent/polymer solutions; 12 kV was identified as the average voltage needed to generate a stable flow of uniform microscale droplets, and 24 kV represented the highest voltage achievable before extensive corona discharge and arcing occurred [23,32]. For sham treatments, the power supply was not turned on and no voltage was applied. A schematic of the electrospraying rig can be seen in Figure 1.

### 2.3. Measuring Viral Infectivity

Immortalized human alveolar adenocarcinomic cells (A-549s) were grown in Dulbecco’s modified Eagle medium (DMEM) with 10% fetal bovine serum (FBS) and 1% penicillin/streptomycin (P/S). Immortalized Chinese hamster ovarian cells (CHOK1s) were grown in Ham’s F-12 medium with 10% FBS. Cells were grown in a temperature- and CO2-controlled incubator at 37 °C and 5% carbon dioxide (CO_2_). Cells were passaged every three days at approximately 80% confluence. In preparation for transduction by either Ad or MVA, A-549 cells were seeded on a 96-well plate at a concentration of 10^4^ cells per well and left to incubate at 37 °C, 5% CO_2_ for 24 h before any virus was added. Ad infectivity after electrospraying was assessed by removing all media from the wells and subsequently adding a solution containing a mixture of electrosprayed and/or solvent exposed Ad in PBS and DMEM such that the multiplicity of infection (MOI) was either 10:1, 100:1, or 1000:1. The same was done in the case of MVA, but the MOI employed was either 0.01:1, 0.1:1, or 1:1. Cells were then left to incubate again for 24 h at 37 °C, 5% CO_2_. To expose the luciferase contained intracellularly, media was replaced with the lysis buffer solution, and cells were left overnight at −80 °C and thawed the next morning. In a white, opaque 96-well plate, lysed cell solution was added followed by an equal amount of luciferase reagent. The resulting luminescence was measured using a microplate reader (FLUOstar Omega, BMG Labtech, Ortenberg, Germany). To express luminescence intensity as a function of total protein content, a BCA assay was also performed to quantify the amount of protein in each well.

### 2.4. Organic Solvent Compatibility

Ten microlitres of concentrated MVA or Ad in PBS were added to 90 µL of either PBS (sham treatment), triacitin, anisole, benzyl acetate, PPh GE, PC, DCM, or toluene in a sterile vial. The resulting immiscible solution was vortexed (Digital Vortex Mixer, VWR, Lutterworth, UK) at 1000 rpm for five seconds. The vial was then left to rest for 60 s, and 400 µL of PBS were added to the vial and 100 µL of the aqueous phase were subsequently transferred to a sterile Eppendorf tube. This 100 µL aliquot was then used to complete a luciferase assay. While all the organic solvents used are immiscible with water, each solvent is marginally soluble in water to varying degrees. To confirm that any potential small quantity of solvent contained in the 100 µL aliquot was inconsequential, the experiment was performed without any viral vectors, and a MTS assay was performed to determine cell viability 24 h after a portion of the aliquot was added to cells.

### 2.5. Statistical Analysis 

The mean average and standard deviation of the luminescence readings were calculated. Two-way ANOVA and Tukey–Kramer or Games–Howell’s post-hoc tests were used to make statistical comparisons on the log transformed data (indicated in the figure captions). Analyses were performed using PRISM 6 (GraphPad Software, San Diego, CA, USA).

## 3. Results and Discussion

### 3.1. Effect of Viral Titre 

Using an appropriate viral titre is vital to generating reliable results. If the titre is too high, the virus may saturate cells as the machinery available for viral infection cannot reasonably accommodate numerous co-infections, and it could be postulated that any reduction in infectivity as a result of exposure to processing conditions may be attenuated or lost completely [33,34]. Operating at too low a titre could significantly increase the variability across samples as some wells may receive no active virus. To ensure an appropriate viral titre range could be established and to examine any effect that concentration may have on resilience to electrospraying, both MVA and Ad were electrosprayed at the highest experimentally relevant voltage, 12 kV, at various titres represented in MOI.

**Figure 2:**
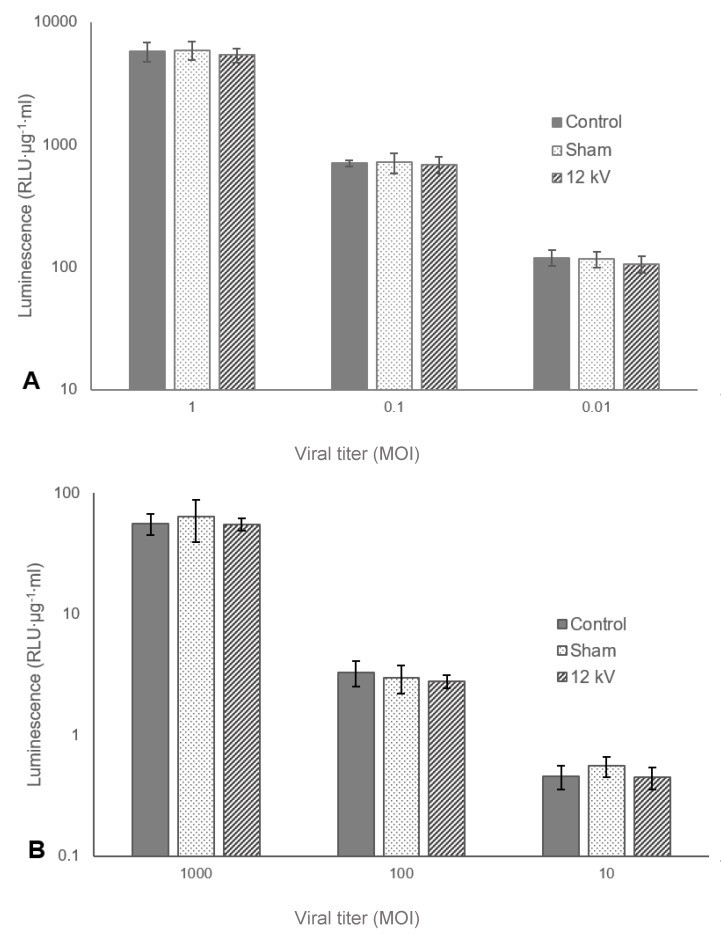
Mean average luminescence, represented as relative light units (RLU) adjusted for protein concentration, measured after lysis of cells incubated with either electrosprayed modified vaccinia Ankara (MVA) (**A**) or adenovirus (Ad) (**B**) at three multiplicities of infection (MOIs). Working distance: 7.25cm, flow rate: 10 µL/min. Error bars represent the standard deviation (*n* = 3).

The results shown in Figure 2A,B indicate respectively that the concentrations of both MVA and Ad were within the appropriate range and neither oversaturating nor undersaturating the plated cells. In both cases, an order of magnitude increase in MOI generated an order of magnitude increase in luminescence. Additionally, within each concentration, each treatment did not differ significantly from any other. Finally, the results indicate that there is a predictable linear relationship between the concentration of infective viral units and luminescence, and for the remainder of the discussion, viral infectivity will be directly inferred from luminescence values.

### 3.2. Effect of Voltage

Establishing an electric field of several thousand volts is typically essential to forming droplets in electrospraying. As indicated in the introduction, however, reactive oxygen species (ROS) generation and/or irreversible electroporation could potentially affect viral infectivity. Hence, it was important to assess the impact of operating voltage upon infectivity and to understand the mechanism of any inactivation. MVA and Ad suspensions were electrosprayed at a range of voltages, with or without the addition of 1 mM of rGSH. rGSH was used as it has been shown in previous studies to reduce the inactivation rate of the bacteriophage MS2 when exposed to an electric current [35]. 

**Figure 3:**
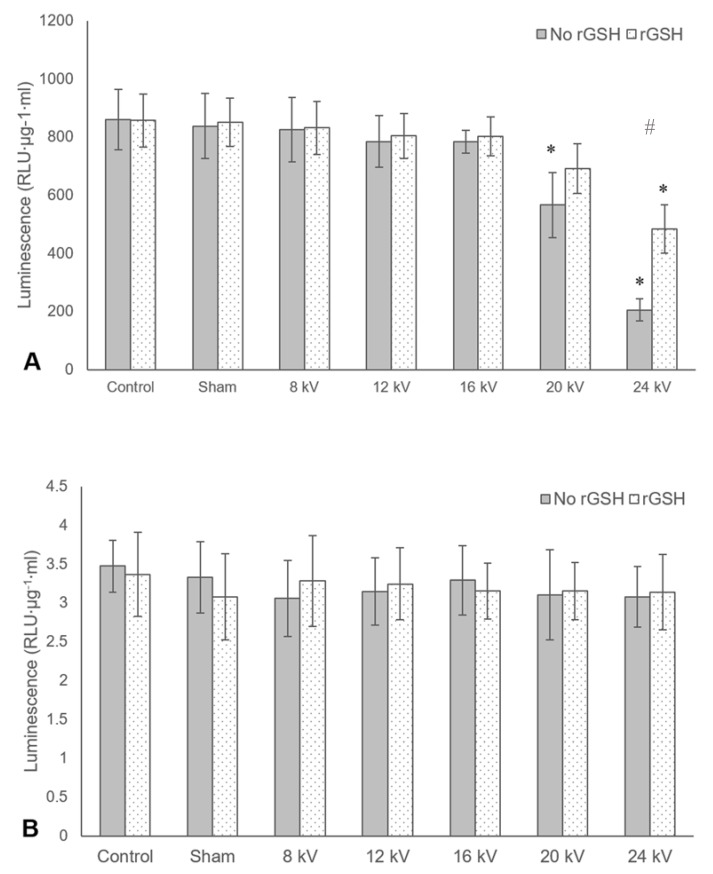
Mean average luminescence, represented as relative light units adjusted for protein concentration, measured after lysis of cells incubated with either electrosprayed MVA (**A**) or Ad (**B**) with or without the presence of rGSH. Working distance: 7.25 cm, flow rate: 10 µL/min. Error bars represent the standard deviation, *n* = 3. Two-way ANOVA and Tukey–Kramer post-hoc tests were used to make statistical comparisons. A *p*-value of less than 0.05 was considered statistically significant. A single asterisk indicates significance compared to respective group control, a hash symbol indicates significance within a given voltage.

Figure 3A shows that without rGSH, MVA infectivity decreased significantly compared to the control at both 20 and 24 kV, but with rGSH, a significant reduction was only seen at 24 kV. Additionally, at 24 kV, MVA without rGSH was significantly less active than MVA with rGSH. Figure 3B, in contrast, indicates that Ad was tolerant across all of the electrospraying conditions tested. One of the main differences between MVA and Ad is the presence of a lipid envelope surrounding the virus. 

The observed reduction in MVA infectivity at high voltages would therefore suggest that irreversible electroporation is likely the main route of inactivation, and the protection afforded by rGSH implies that pore formation allows for exacerbated damage from ROS generated during electrospraying. It is important to note that the relatively large diameter of MVA also likely reduces its tolerance to electrospraying. Evidence of smaller enveloped viruses exhibiting greater resistance to electric fields can be found in the study conducted by Mizuno et al., where 1.33 to 2.67 times as many 30 kV pulses were required to inactivate swine vesicular disease virus (SVDV) compared to herpesvirus-1, which is more than twice the diameter of SVDV [36].

### 3.3. Organic Solvent Compatibility

Ad and MVA were exposed to seven solvents to better understand how each solvent impacted the infectivity of the virus. Triacetin, anisole, benzyl acetate, PPh GE, and PC were selected for their amenable electrospraying properties and favourable toxicity profiles. DCM and toluene were selected for their prevalence in a variety of encapsulation techniques [37,38,39]. A separate experiment was also performed to check whether any trace solvent in the virus suspensions would affect the viability of the A549 cells. No difference in cell viability was found across any of the samples exposed to solvents only. 

**Figure 4:**
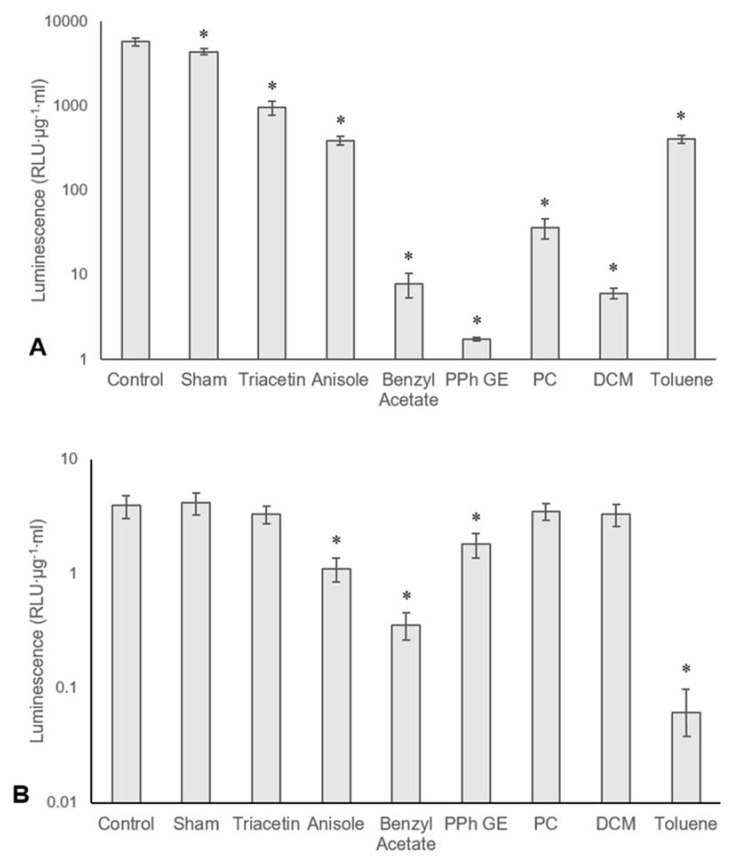
Mean average luminescence, represented as relative light units adjusted for protein concentration, measured after lysis of cells incubated with solvent-exposed MVA (**A**) or Ad (**B**). Welch’s ANOVA and Games–Howell’s post-hoc tests were used to make statistical comparisons. A *p*-value of less than 0.05 was considered statistically significant and indicated by an asterisk. Error bars represent the standard deviation, *n* = 3.

In the case of MVA (Figure 4A), there was a significant difference in infectivity between each of the solvent groups. Triacetin was found to be the least damaging, producing an 83.4% (SD ± 3.3%) decrease in infectivity compared to the control. Benzyl acetate, PPh GE, PC, and DCM were the most harmful, all reducing infectivity by more than 99%. Interestingly, the sham treatment of vortexing without the addition of solvent also caused a significant decrease in infectivity (23.8%, SD ± 6.1%), suggesting that MVA may be susceptible mechanical stress. Ad (Figure 4B) demonstrated greater stability to solvent exposure, as only anisole, benzyl acetate, PPh GE, and Toluene caused a significant decrease in infectivity. 

The fact that MVA was affected more severely by solvent exposure is again likely due to its being enveloped. As above, solvents and detergents are routinely used to inactivate lipid-enveloped viruses [31] but typically have an insignificant effect on non-lipid enveloped viruses [40]. These results confirm that solvent selection may be extremely important in enabling viral encapsulation. This would be true not only for electrospraying but any technique requiring the use of polymer solutions. Indeed, techniques such as double emulsion solvent evaporation/extraction involve more extensive solvent exposure. 

### 3.4. Coaxial Electrospray with Organic Solvent

MVA and Ad were not shown to be negatively affected by single needle electrospraying at experimentally relevant voltages. Encapsulation within core shell particles is, however, only possible with coaxial electrospraying [41]. Hence, to determine the effects of the electric field and organic solvent exposure during coaxial electrospraying, a solution of pure DCM was passed through the outer needle and coaxially electrosprayed with an inner solution of MVA or Ad. Due to its particularly volatile nature, it was assumed that DCM would not remain as a contaminant after electrospraying [23,32,42].

**Figure 5:**
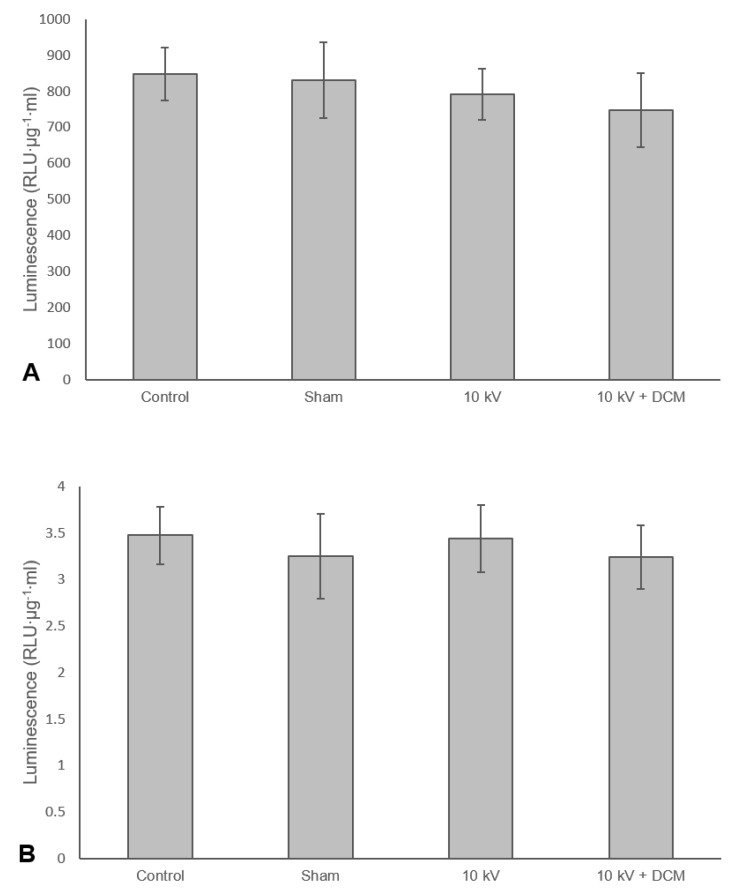
Luminescence, represented as relative light units adjusted for protein concentration, measured after lysis of cells incubated with either MVA (**A**) or Ad (**B**) after single or coaxial electrospray. Working distance: 7.25 cm, Virus solution flow rate: 5 µL/min, dichloromethane (DCM) flow rate: 25 uL/min. One-way ANOVA and Tukey’s post-hoc tests were used to make statistical comparisons. A *p*-value of less than 0.05 was considered statistically significant. Error bars represent the standard deviation, *n* = 3.

In contrast to the results shown in Figure 4, there was no significant reduction in infectivity of either MVA (Figure 5A) or Ad (Figure 5B) when subjected to coaxial electrospraying at 10 kV. These results suggest that it is not only the solvent but also the contact time that is important and that the latter is sufficiently short during electrospraying to limit the effect on viral infectivity. This is a further potential advantage over either conventional emulsification or microfluidic techniques, both of which involve more prolonged solvent exposure. DCM in particular offers the further advantage of rapid particle formation and good biocompatibility, again due to its high volatility. 

## 4. Conclusions

The aim of this study was to investigate the feasibility of using electrospraying for virus encapsulation. In particular, it was sought to determine whether the high electric fields and organic solvents typically used in electrospraying would damage the infectivity of two common therapeutic viruses, MVA and Ad. The results indicate that neither virus was adversely affected under practically relevant processing conditions, up to maximum voltage of 16 kV. The infectivity of Ad was not compromised at any of the voltages tested. The infectivity of MVA was compromised at 20 kV and above. This was attributed to it having a lipid envelope which, unlike Ad, made it susceptible to electroporation and damage by ROS. It was found that adding the ROS scavenger rGSH had a significant protective effect. MVA also suffered significant reductions in infectivity when exposed directly to all of the solvents tested in this study. The effect was particularly pronounced with DCM, PPh GE, and benzyl acetate. Again, this was attributed to it being enveloped. 

When MVA was co-axially electrosprayed with DCM, however, its infectivity was preserved. This suggests that the effect of a solvent is dependent upon exposure time and that this is sufficiently short during coaxial electrospraying to minimize any damage. Ad was found to be robust to all electrospraying and co-electrospraying configurations tested and was only inactivated to a significant degree when exposed directly to anisole, benzyl acetate, PPh GE, and toluene. 

The results presented thus further support the development of coaxial electrospraying as a promising technique for encapsulating complex and sensitive biological material. Future work will investigate its application for compound vaccine development and delivery of oncolytic viruses.
